# Mitochondrial heteroplasmy as a cause of cell-to-cell phenotypic heterogeneity in clonal populations

**DOI:** 10.3389/fcell.2023.1276629

**Published:** 2023-10-09

**Authors:** Dmitry A. Knorre

**Affiliations:** Department of Molecular Energetics of Microorganisms, Belozersky Institute of Physico-Chemical Biology, Lomonosov Moscow State University, Moscow, Russia

**Keywords:** yeast, heterogeneity, heteroplasmy, pathogenicity threshold, mtDNA, retrograde signaling, mitochondrial dysfuncion

## Introduction

Individual cells within a clonal population can display pronounced phenotypic differences. In multicellular organisms, such differentiation is controlled by numerous microenvironmental, positional, and genetic cues. However, even unicellular *Saccharomyces cerevisiae* cells show significant levels of cell-to-cell variability. This variability arises from *de novo* mutations, gene expression noise and asymmetric division. Here, I suggest that another contributing factor could be genetic drift in mitochondrial DNA (mtDNA) heteroplasmy levels. Moreover, because of the activation of general stress response pathways in cells with insufficient levels of wild type mtDNA, heteroplasmic cells can withstand certain stresses better than wild type cells. As a result, the heterogeneity within the heteroplasmic cell population could be a bet-hedging mechanism, increasing the chances of cell line survival.

## Main text

Eukaryotic cells reproduce asexually most of the time. In the species with obligate sexual reproduction, there are thousands of mitotic divisions per meiotic division. For instance, human body comprises 3*10^13^ clonal somatic cells (Sender et al., 2016) that correspond to at average 13.4 divisions of zygote descendants. The actual number of cell generations is even higher due to cell turnover. Moreover, certain species have facultative sexual reproduction, so baker’s yeast cells go through a sexual reproduction phase only once in every ∼10,000 generations (Nieuwenhuis and James, 2016). Therefore, a majority of the living eukaryotic cells have multiple clonal copies of their own.

Clonal cells, despite their genetic identity, can pronouncedly differ from each other by the phenotype. This is true, even in the case of unicellular organisms, such as baker’s yeast *S. cerevisiae.* This is evidenced by differing protein concentrations and stress response readiness in genetically identical cells (Levy et al., 2012). The driving forces behind this heterogeneity include gene expression noise, cyclic processes, and asymmetry in cell divisions (Knorre et al., 2018). Recent research has shown that molecular stochasticity of 5′-UTR scanning by ribosome contributes to yeast population heterogeneity in cell response to nutrient limitation (Meng et al., 2023). In certain cases, yeast cells suspensions exhibit phenotypic bistability which is characterised by the coexistence of cells with two distinguishable phenotypes in a clonal cell line. For example, introns mediate bimodal distribution of the yeast ribosomal protein Rps22B in yeast cells (Lukačišin et al., 2022).

Cellular regulatory systems serve to minimise phenotypic variation. Therefore, gene promoter regions are subject to purifying selection pressure which mitigates gene expression noise (Metzger et al., 2015). Indeed, an elevation in phenotypic variance means that a growing percentage of cells within a subpopulation deviates from an optimal phenotype. However, in some cases there is no single optimal phenotypic value, particularly when the optimal protein concentration within a cell depends on fluctuating environmental conditions. In particular, the growth rate of yeast negatively correlates with its cellular resistance to stress (Ho and Gasch, 2015). At the same time, a genetically homogeneous suspension culture of yeast is usually heterogeneous in the rate of division of individual cells (Arabaciyan et al., 2021) where slow-dividing cells upregulate general stress-response transcription factors Msn2p/Msn4p and exhibit a higher trehalose synthase Tsl1 concentration (Li et al., 2018). The high trehalose synthase Tsl1p concentration is beneficial during heat stress but associated with decreased growth rate under normal conditions (Levy et al., 2012). We have also recently demonstrated that post-diauxic yeast cells form two subpopulations characterised by high and low concentrations of mitochondrial ATP-ase inhibitor proteins (Inh1p and Stf1p). Cells with an elevated inhibitor level recovered more rapidly from the stationary phase than their low-level counterparts, at the same time a high concentration of the inhibitor proteins was deleterious under the conditions of mitochondrial dysfunction (Galkina et al., 2022). These examples show that cell culture phenotypic heterogeneity can be detrimental or adaptive depending on which specific phenotypic properties are considered.

Eukaryotic cells harbour mitochondria, the cellular organelle with its own genome; refer to (Janouškovec et al., 2017) for the rare exceptions. The number of mtDNA molecules in cells varies depending on conditions, cell type and the previous cell’s history (Medeiros et al., 2018; Zhang et al., 2021; Galeota-Sprung et al., 2022). Meanwhile, heteroplasmy, a condition characterised by the presence of multiple mtDNA variants within a single cell, may occur as a result of mutations or cell fusion (Figure). The heteroplasmy is a common phenomenon. Many human genetic mitochondrial disorders are linked to the coexistence of a wild type mtDNA molecule and a variant bearing a deleterious mutation (Wallace and Chalkia, 2013). Furthermore, heteroplasmy in germline can be transmitted for several generations (Burgstaller et al., 2018).

Several forces shape mtDNA allele frequency within a cell line (Rand, 2001). 1) The random sampling of mtDNA during cell division makes the relative number of two mitochondrial genotypes drift over generations (Figure). The drift continues until one of the variants becomes fixed. Given that mtDNA can replicate under cell cycle arrest (Newlon and Fangman, 1975), a proportion of different mtDNAs can also change over time in post-mitotic heteroplasmic cells (Taylor and Turnbull, 2005). 2) At the same time, dividing cells with a high proportion of the pathogenic mtDNA molecules become unable to proliferate and, therefore, they are outcompeted by other cells with functional mitochondria. As accumulation of mutant mtDNA in a cell has a threshold effect on a cell phenotype (Rossignol et al., 2003), pathogenic mtDNA variants can persist in a cell line at a low proportion for an extended period. The pathogenicity threshold usually lies in the range 50%–90% of mutant mtDNA allele frequency; the number depends on cell type and the nature of mutation (Rossignol et al., 2003). This suggests that the wild type mtDNA is usually present in a moderate excess in normal cells. 3) Due to their superior intracellular fitness, certain deleterious mtDNAs variants can displace wild type variants within the cell (Gitschlag et al., 2020).

These three factors contribute to the ability of harmful mtDNA variants to persist in clonal populations along with wild type mtDNAs. Such populations should sporadically produce cells with a high proportion of mutant mtDNA and dysfunctional mitochondria. Indeed, multicellular organisms develop from a single cell, which experiences a significant genetic bottleneck in mtDNA quantity. This bottleneck decreases the diversity of mtDNA variants (Krakauer and Mira, 1999; Zaidi et al., 2019). Despite this, the proportion of respiration-deficient cells in some tissues increases with age (Taylor and Turnbull, 2005; Payne et al., 2011; Hipps et al., 2022).

Eukaryotic cells are equipped with the mechanisms capable of compensating deleterious effects of mitochondrial dysfunction. In nematodes, for instance, the depletion of mitochondrial DNA triggers the expression of mitochondrial chaperones (Nargund et al., 2012). In addition, mitochondrial dysfunction induces the general stress response mechanisms, which encompass both mitochondrial and cytosolic heat-shock response proteins (Poveda-Huertes et al., 2020). Furthermore, yeast cells with dysfunctional mitochondria demonstrate a surprising resilience to certain stress factors, outperforming cells with functional oxidative phosphorylation. Such yeast cells display enhanced resistance to a range of stressors, including the protein synthesis inhibitor cycloheximide (Zhang and Moye-Rowley, 2001), cytoplasm acidification (Ludovico et al., 2002), mating pheromone-induced death (Severin and Hyman, 2002), lipophilic cations (Antonenko et al., 2011), and the prooxidant paraquat (Stenberg et al., 2022). The resistance to these stressors is facilitated, in part, by the activation of pleiotropic drug resistance (PDR) transporter genes in yeast cells without functional mtDNA. These genes encode ABC-transporters with broad substrate specificity capable of effluxing xenobiotics from the cytoplasm into the environment (Zhang and Moye-Rowley, 2001).

**FIGURE 1:**
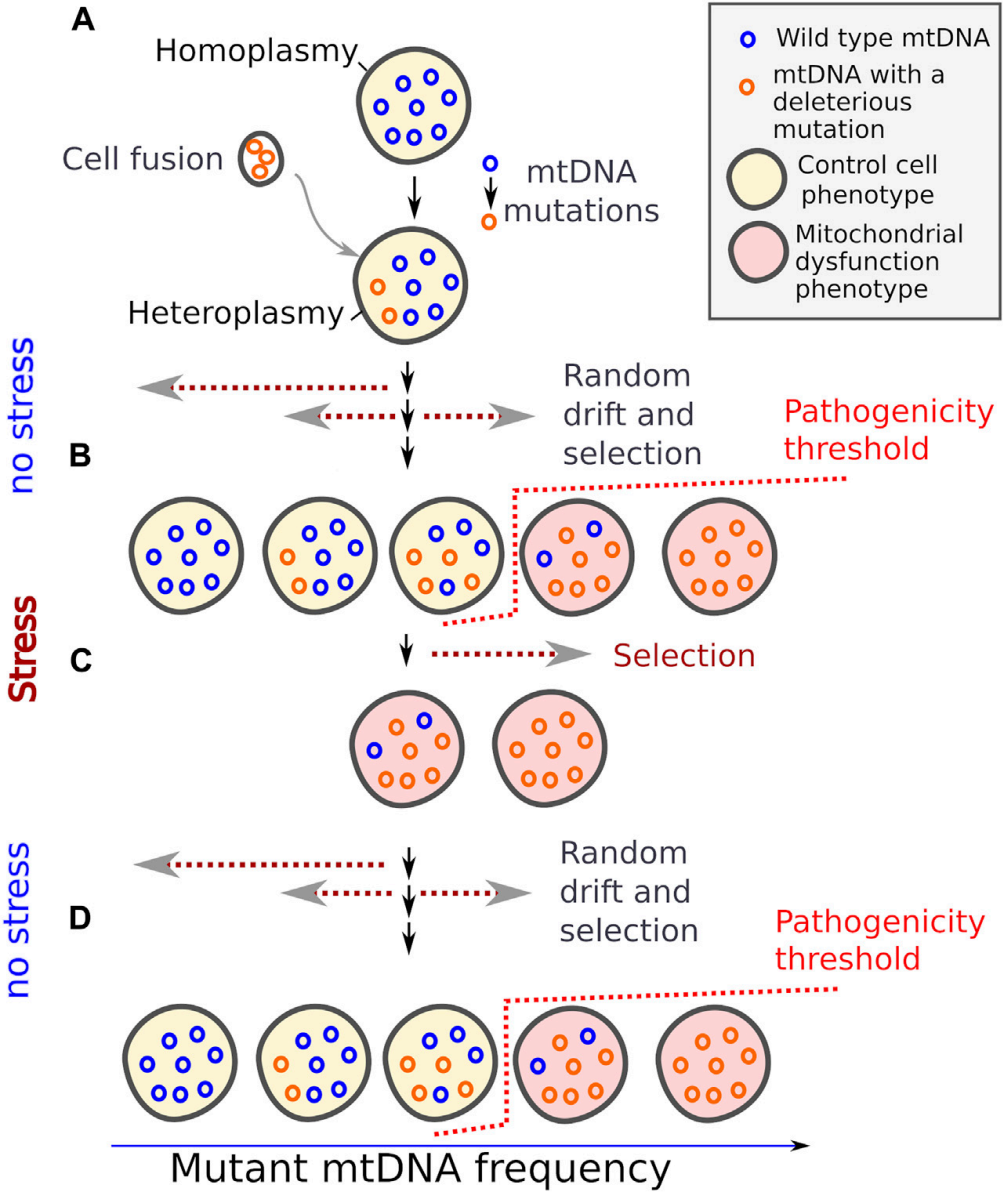
Mitochondrial DNA heteroplasmy provides bistability in mitochondrial dysfunction phenotypes. Random mutations and genetic drift generate a fraction of cells with dysfunctional mitochondria **(A)**. These cells are outcompeted by the cells with low burden of mutant mtDNA under normal conditions **(B)**, but better survive under certain stresses **(C)**, and wild type mtDNA can repopulate cells again when the stress is relieved. **(D)** Dotted arrows indicate the direction of change in mtDNA allele frequencies.

Taken together, it turns out that, on the one hand, in clonal populations mitochondrial heteroplasmy causes the sporadic occurrence of the cells with impaired mitochondrial function; and mitochondrial dysfunction activates protective mechanisms. On the other hand, the activation of such a mechanism can be useful for cells in a wide range of stressful conditions. Importantly, in a heteroplasmic cell with a high load of mutant mtDNA but retaining wild-type mtDNA the proportion of full-length wild-type mtDNA can be increased over several generations in the absence of stress. Therefore, mitochondrial heteroplasmy can be a natural way to generate phenotypic bistability in clonal populations. I suggest that bistability mediated by mitochondrial heteroplasmy can be a mechanism betting the risk in the fluctuating environment (Figure 1).
